# Comparative Analysis of Clinical Outcomes in High-Grade Glioma Patients: 5-ALA Fluorescence-Guided Surgery vs. Conventional White-Light Resection

**DOI:** 10.3390/cancers17121897

**Published:** 2025-06-06

**Authors:** Nurzhan Ryskeldiyev, Aidos Moldabekov, Dinara Berdibayeva, Aiman Maidan, Torebek Tursynbekov, Dimash Davletov, Muratbek Tleubergenov, Assel Kabykenova, Diana Kerimbayeva, Aidos Doskaliyev, Serik Akshulakov

**Affiliations:** 1 National Centre for Neurosurgery, Astana 010000, Kazakhstan; nurjan.ryskeldiev@ncn.kz (N.R.); aidos.moldabekov@ncn.kz (A.M.); dinara.berdibaeva@ncn.kz (D.B.); torebektursynbekov@gmail.com (T.T.); muratbek.tleubergenov@ncn.kz (M.T.); kabykenova.ad@ncn.kz (A.K.); aidos.doskaliev@ncn.kz (A.D.); serik.akshulakov@ncn.kz (S.A.); 2 Taraz City Multidisciplinary Hospital and Consulting and Diagnostic Center, Taraz 080000, Kazakhstan; 3 Atchabarov Scientific-Research Institute of Fundamental and Applied Medicine, Asfendiyarov Kazakh National Medical University, Almaty 050000, Kazakhstan; 4 Department of Psychology/Neuroscience Program, Dickinson College, Carlisle, PA 17013, USA; kerimbad@dickinson.edu

**Keywords:** 5-aminolevulinic acid, glioblastoma, fluorescence-guided surgery, extent of resection (EOR), neuro-oncology

## Abstract

Background High-grade gliomas (HGGs) are aggressive brain tumors with poor prognoses. Maximizing the extent of resection (EOR) is a critical surgical goal. Fluorescence-guided surgery using 5-aminolevulinic acid (5-ALA) has been proposed to enhance tumor visualization and resection. MethodsWe retrospectively analyzed 141 patients with histologically confirmed HGGs who underwent either 5-ALA-guided (*n* = 71) or conventional white-light (*n* = 70) resection between 2018 and 2023. Propensity score matching and multivariate Cox regression models were used to assess the impact of 5-ALA on surgical outcomes and survival. Results: Gross total resection (GTR) was significantly more common in the 5-ALA group than the conventional white-light group (28.17% vs. 12.86%, *p* = 0.0245). Kaplan–Meier analysis showed no statistically significant difference in overall survival between groups after matching (log-rank *p* = 0.6371). However, patients with GTR had significantly improved survival compared to those with subtotal resection (log-rank *p* = 0.0423). Multivariate Cox regression identified radiotherapy (HR = 0.291, 95% CI: 0.166–0.513, *p* < 0.001), higher Karnofsky Performance Status (HR = 0.962, 95% CI: 0.942–0.982, *p* = 0.0003), and GTR (HR = 0.476, 95% CI: 0.272–0.834, *p* = 0.0091) as independent predictors of improved survival. 5-ALA usage was not an independent predictor (HR = 0.885, 95% CI: 0.554–1.413, *p* = 0.612). Radiotherapy and chemotherapy were more frequently administered in the conventional white-light group (*p* = 0.0404 and *p* = 0.0085, respectively). Conclusions 5-ALA fluorescence-guided surgery significantly increases the rate of gross total resection in high-grade glioma patients but does not independently confer a survival advantage. Survival outcomes are primarily influenced by the extent of resection, adjuvant therapy, and functional status. Integration of 5-ALA within a comprehensive oncological framework may enhance its clinical utility.

## Simple Summary

Brain tumors known as high-grade gliomas are difficult to treat because they grow quickly and are hard to remove completely. A special technique using a compound called 5-ALA helps surgeons better see the tumor during surgery by making cancer cells glow under blue light. This study looked at how patients who had surgery with this glowing technique compared to those who had regular surgery without it. We found that using 5-ALA helped surgeons remove more of the tumor but did not clearly improve survival when other treatments and factors were considered. Our findings suggest that while this technique can help achieve more complete tumor removal, its full benefit may depend on additional treatments like radiotherapy and the overall health of the patient. These results may help guide future research and improve how we use advanced surgical tools in real-world hospital settings.

## 1. Introduction

High-grade gliomas encompass a heterogeneous collection of aggressive primary brain tumors, including glioblastoma, which exhibit rapid growth, high rates of recurrence, and dismal prognosis [1]. The classification of gliomas has evolved significantly over the past century, from a purely histological approach to an integrated system combining both morphological and molecular features. As outlined in the 2021 WHO Classification of Central Nervous System Tumors, adult-type diffuse gliomas are now stratified based on key molecular markers, particularly isocitrate dehydrogenase (IDH) mutation, TERT promoter mutation, and chromosome 7 gain/10 loss, in addition to histopathologic criteria [2]. Among these, IDH-wildtype glioblastoma (GBM) represents the most aggressive and prevalent subtype, classified as WHO Grade 4. It is defined not only by histological hallmarks such as microvascular proliferation or necrosis, but also by specific molecular alterations, including EGFR amplification and TP53/ATRX wild-type status. These molecular insights have enhanced our understanding of glioma behavior and prognosis, underscoring the necessity of integrating genomic profiling into surgical and therapeutic planning [2].

Despite advances in surgical methods, chemotherapy, and radiotherapy, the overall survival of patients with high-grade gliomas remains poor, with a median survival of approximately 15 months for glioblastoma and slightly longer for other high-grade glioma subtypes [3,4]. Maximizing the extent of tumor resection is a crucial factor in enhancing survival outcomes, yet complete tumor removal is challenging due to the infiltrative nature of these tumors and the difficulty in differentiating malignant tissue from healthy brain parenchyma under standard white-light visualization [5].

The introduction of fluorescence-guided surgery using 5-aminolevulinic acid (5-ALA), marketed as Gliolan, has significantly impacted the field of neuro-oncology by enhancing the surgeon’s ability to visualize and resect malignant tissues more accurately. 5-ALA is a precursor to protoporphyrin IX, a photosensitive compound that accumulates selectively in tumor cells, causing them to fluoresce under blue light. Numerous studies, including randomized controlled trials and large cohort studies, have demonstrated that the use of 5-ALA significantly increases the EOR, leading to better progression-free survival (PFS) and overall survival rates in patients with high-grade gliomas [6].

Stummer et al. [7] conducted a landmark phase III trial that established the efficacy of 5-ALA in improving the EOR and reducing residual tumor volume, which in turn correlated with prolonged survival. More recent studies, such as the one by Mirza et al. [8], have further confirmed these findings, demonstrating improved OS and reduced neurological deficits in patients undergoing 5-ALA-guided resection compared to those undergoing traditional white-light surgery. These studies highlight the growing evidence that fluorescence-guided surgery is becoming a standard of care in high-grade glioma management.

However, while the benefits of 5-ALA-guided surgery are well-documented, comparative data between patients undergoing 5-ALA-assisted surgery and those undergoing conventional resection without 5-ALA are still limited, especially in specific regional contexts. This study aims to fill this gap by comparing two groups of high-grade glioma patients treated at our center between January 2022 and December 2023: one group receiving 5-ALA for fluorescence-guided resection and a second group undergoing standard white-light surgery.

The findings from this study could offer valuable information for neurosurgeons and healthcare providers regarding the adoption of fluorescence-guided surgery as a standard practice in high-grade glioma management. Furthermore, this study seeks to explore the practical implications of using 5-ALA in a real-world setting, addressing potential challenges and benefits that could guide future clinical decisions and research.

## 2. Materials and Methods

### 2.1. The Study Design

This retrospective study included adult patients with histologically confirmed high-grade gliomas who underwent surgical resection at a tertiary care center between January 2022 and December 2023.

### 2.2. Patient Selection

Patients included in the study were diagnosed with high-grade gliomas, confirmed through histopathological analysis. Inclusion criteria were age ≥ 18 years, histopathologically confirmed high-grade tumor, and complete preoperative and postoperative imaging and clinical data availability. Exclusion criteria included patients with non-glial tumors, incomplete medical records, or those who underwent biopsy without resection.

Inclusion criteria were a contrast-enhancing mass suggestive of high-grade glioma (HGG) on preoperative T1-weighted magnetic resonance imaging (MRI), potential resectability of the lesion, early postoperative contrast-enhanced MRI for volumetric analysis, complete datasets on adjuvant treatment, and comprehensive radiographic and clinical follow-up as determined by the interdisciplinary tumor board. Final histopathological diagnosis had to be either glioblastoma, IDH-wildtype, CNS WHO Grade 4 or astrocytoma, IDH-mutant, CNS WHO Grade 4, the latter defined according to the 2021 WHO classification as IDH1-mutant tumors with histological features such as necrosis and/or microvascular proliferation. We excluded cases of recurrent HGG, low-grade gliomas (WHO Grade I or II), and patients lost to follow-up. The conventional white-light group represented a historical cohort with comparable tumor location and volume characteristics. A lesion was defined as being “eloquent” if it was located within or adjacent to an eloquent area, thus potentially interfering with the resection of more than the contrast-enhancing tumor. The location “other” was used for subcortical eloquent areas like the corpus callosum, basal ganglia, and brainstem. An EoR > 95% was defined as a GTR. Progression-free survival (PFS) was defined as survival from the last surgery until the first radiological description of a progression. Follow-up MRI was routinely performed every 3 months. Decision making was based on the Response Assessment in Neuro-Oncology criteria (RANO) as actualized in 2010 [9]. Overall survival (OS) was defined as survival after the date of primary diagnosis in months. New permanent neurological deficits were defined as neurological deficits not described prior to surgery that were still eminent at the first follow-up assessment (3 months). MGMT promotor methylation was assessed and reported according to Hegi et al. [10].

### 2.3. 5-ALA Administration and Surgical Procedure

Prior to surgery, patients in the 5-ALA group received an oral dose of 5-ALA (20 mg/kg body weight) approximately 3 to 4 h before the planned craniotomy. The 5-ALA was administered under the supervision of a neurosurgical team to ensure optimal timing and compliance. The surgical procedures were performed using a ZEISS BLUE 400 surgical microscope (Zeiss, Oberkochen, Germany) equipped with fluorescence filters to detect tumor tissue that accumulated protoporphyrin IX, resulting in visible fluorescence under blue light.

In the conventional white-light group, standard white-light surgical resection techniques were used without the aid of fluorescence guidance.

### 2.4. Imaging and Intraoperative Monitoring

All patients underwent preoperative and early postoperative contrast-enhanced MRI. Gross total resection (GTR) was defined as complete removal of the contrast-enhancing tumor on early postoperative MRI, typically within 72 h. Subtotal resection (STR) indicated any residual enhancement. Tumor location was categorized anatomically (frontal, temporal, parietal, occipital, or deep structures such as corpus callosum, basal ganglia, brainstem). Tumors in or near the eloquent cortex were flagged based on preoperative imaging.

### 2.5. Data Collection

Patient demographics, clinical characteristics, and surgical details were collected from medical records. Data points included age, gender, Karnofsky Performance Status (KPS), tumor volume, extent of resection (gross total or subtotal), and postoperative neurological outcomes. The two groups were compared to assess the impact of 5-ALA on surgical outcomes

### 2.6. Outcome Measures

The primary outcome measure was the extent of resection (EOR), defined as gross total resection (GTR) when no residual contrast-enhancing tumor was observed on postoperative MRI. Secondary outcome measures included progression-free survival (PFS), overall survival (OS), and changes in neurological function post-surgery, evaluated using the KPS scale.

### 2.7. Statistical Analysis

Statistical analysis and visualizations were made in SAS University Edition, version 3.8 (SAS Institute Inc., Cary, NC, USA) and Python 3.12 (Python Software Foundation, Beaverton, OR, USA). Descriptive statistics were used to summarize patient demographics and clinical characteristics. Categorical variables were presented as frequencies and percentages (n [%]). Comparative analysis between the two groups was performed using chi-squared and Fisher’s exact tests for categorical variables. Moreover, McNemar’s test was utilized for categorical variables in paired analyses. Kaplan–Meier survival curves and log-rank tests were used to assess the overall survival in various groups. Unadjusted and adjusted Cox proportional hazard regression models were used to evaluate the impact of different conditions on the overall survival. In addition, the association of a history of 5-ALA versus the conventional white-light group with all-cause mortality was evaluated after 1:1 propensity score matching with the covariates from the regression model in a Kaplan–Meier curve. A *p*-value of <0.05 was considered statistically significant. The Karnofsky Performance Scale was transformed into categorical data by grouping at a cut-off point of 70%, as it represents the division point between a person’s ability to carry on normal activity and work without special assistance.

### 2.8. Ethical Considerations

The institution’s ethics committee approved the use of 5-ALA for fluorescence-guided surgery on 15 April 2021. Data collection adhered to patient confidentiality protocols, and all data were anonymized before analysis.

## 3. Results

The analysis included 141 patients, with 71 patients in the 5-ALA group and 70 in the conventional white-light resection group. The dataset of the 5-ALA group had a male-to-female ratio of 44:50. The mean age at presentation was 48.8 years, with an age range of 18 to 77 years. The most represented regions were Astana (16 patients), Zhambyl Region/Taraz (4 patients), Akmola Region/Kokshetau (3 patients), and Mangystau Region/Aktau (3 patients). Baseline characteristics are summarized in Table 1. Detailed patient-level data including 5-ALA and control groups is provided in Supplementary File S1.

**Table 1:** Comorbidities and their influence.

Comorbidities					
Obesity	5-ALA	White-Light Group	Total	*p*-Value	Statistical Test
No	47 (67.14%)	50 (70.42%)	97	0.6743	Chi-square
Yes	23 (32.86%)	21 (29.58%)	44		
**Diabetes Mellitus**					
No	65 (92.86%)	69 (97.18%)	134	0.2748	Fisher’s Exact
Yes	5 (7.14%)	2 (2.82%)	7		
**Ischemic heart disease**					
No	56 (80.00%)	61 (85.92%)	117	0.35	Chi-square
Yes	14 (20.00%)	10 (14.08%)	24		
**Arterial hypertension**					
No	40 (57.14%)	46 (64.79%)	86	0.352	Chi-square
Yes	30 (42.86%)	25 (35.21%)	55		
**EoR**					
Subtotal Resection	61 (87.14%)	51 (71.83%)	112	0.0245	Chi-square
Gross Total Resection	9 (12.86%)	20 (28.17%)	29		
**Event**					
Alive	27 (38.57%)	24 (33.80%)	51	0.5557	Chi-square
Death	43 (61.43%)	47 (66.20%)	90		

### 3.1. Survival and Extent of Resection

Gross total resection (GTR) was significantly more frequent in the 5-ALA group compared to the conventional white-light group (71.25% vs. 28.75%, *p* = 0.0002). Subtotal resection (STR) remained more prevalent in the white-light group (Table 1). The overall survival difference between the groups was non-significant (*p* = 0.2478).

**Figure 1:**
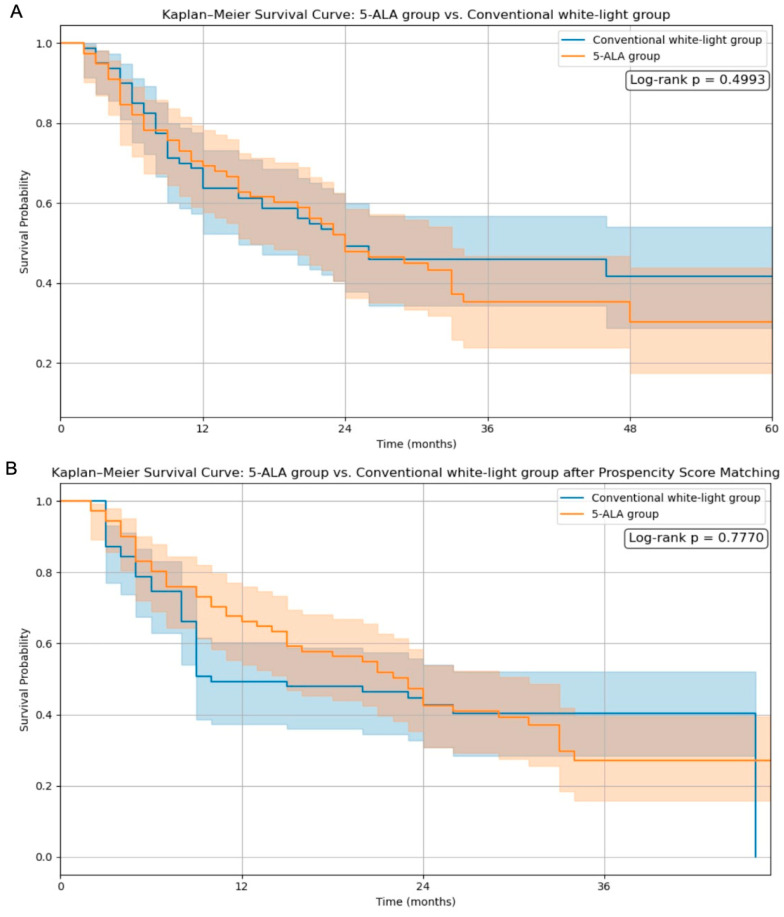
The Kaplan–Meier survival analysis before (**A**) and after (**B**) propensity score matching.

Kaplan–Meier survival curves were analyzed to evaluate the impact of 5-ALA use and the extent of resection on overall survival. As shown in Figure 1A, the comparison between the 5-ALA and conventional white-light groups without matching demonstrated no significant difference in survival (log-rank *p* = 0.4993). Following propensity score matching (Figure 1B), the survival trajectories remained comparable, and the log-rank *p*-value was 0.7770, indicating that 5-ALA use did not confer a statistically significant survival benefit even after adjusting for baseline characteristics.

**Figure 2:**
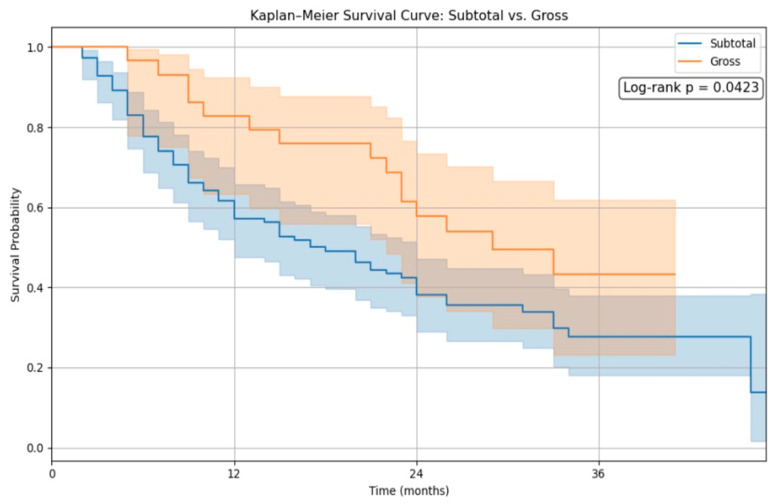
The Kaplan–Meier survival analysis of EoR.

In contrast, Figure 2 illustrates the survival difference between patients who underwent gross total resection (GTR) and those with subtotal resection (STR). The GTR group exhibited significantly improved survival, with a log-rank *p*-value of 0.010. This finding reinforces the prognostic importance of the extent of resection as a critical determinant of survival in high-grade glioma patients.

### 3.2. Postoperative Function and Karnofsky Score

Postoperative KPS scores ≤ 70 were more frequent in the 5-ALA group (67.5%) compared to the conventional white-light group (53.75%), but this difference was not statistically significant (*p* = 0.0865). Tumor eloquence played a role, particularly in tumors involving the parietal lobe and deep brain regions, as seen in Table 2.

### 3.3. Comorbidities and Adjuvant Therapy

In the comparison between patients who received 5-ALA and those who underwent standard white-light resection, no statistically significant differences were observed in survival status (alive vs. deceased; *p* = 0.5557), obesity (*p* = 0.6743), diabetes mellitus (*p* = 0.2748), ischemic heart disease (*p* = 0.3500), or arterial hypertension (*p* = 0.3520). Additionally, no significant differences were found in tumor size, hemisphere (left, right, bilateral), KPS scores (pre- or postoperative), tract involvement (corticospinal, longitudinal, visual, or commissural), re-surgery rates, or postoperative therapy types.

However, significant differences were noted in several key clinical variables. Radiotherapy and chemotherapy were both administered more frequently in the white-light group (radiotherapy: 72.86% vs. 56.34%, *p* = 0.0404; chemotherapy: 65.71% vs. 43.66%, *p* = 0.0085), potentially reflecting better functional status or oncologic eligibility. Gross total resection (GTR) was significantly more common in the 5-ALA group (28.17% vs. 12.86%; *p* = 0.0245), supporting the role of 5-ALA in facilitating more extensive tumor removal.

The location of the tumor also differed between groups (*p* = 0.0154), as seen in Table 3: the 5-ALA group exhibited a higher proportion of tumors located in the temporal (15.49% vs. 2.86%) and parietal (14.08% vs. 7.14%) lobes, while the conventional white-light group had a greater number of tumors categorized as being located in “other” deep or multifocal locations (38.03% vs. 55.71%). Furthermore, IDH1 mutation status varied significantly between groups (*p* = 0.0149), with the 5-ALA cohort showing a higher proportion of IDH1-negative tumors, consistent with more aggressive disease biology.

**Table 3:** Tumor characteristics.

Location	5-ALA	White-Light Group	Total	*p*-Value	Statistical Test
Frontal	24 (34.29%)	21 (29.58%)	45	0.0154	Fisher’s Exact
Temporal	2 (2.86%)	11 (15.49%)	13		
Parietal	5 (7.14%)	10 (14.08%)	15		
Occipital	0 (0.00%)	2 (2.82%)	2		
Others	39 (55.71%)	27 (38.03%)	66		
**Side**					
Left	31 (44.29%)	34 (47.89%)	65	0.5801	Chi-square
Right	28 (40.00%)	30 (42.25%)	58		
Both	11 (15.71%)	7 (9.86%)	18		
**Tumor size**					
Giant	34 (48.57%)	40 (56.34%)	74	0.5124	Fisher’s Exact
Medium	32 (45.71%)	29 (40.85%)	61		
Small	4 (5.71%)	2 (2.82%)	6		
**Radiotherapy**					
No	19 (27.14%)	31 (43.66%)	50	0.0404	Chi-square
Yes	51 (72.86%)	40 (56.34%)	91		
**Chemotherapy**					
No	24 (34.29%)	40 (56.34%)	64	0.0085	Chi-square
Yes	46 (65.71%)	31 (43.66%)	77		

### 3.4. Pre- and Postoperative Karnofsky Performance Scale

The study found that 33% of patients had a postoperative Karnofsky Performance Status (KPS) of over 70, of which 2/3 of these patients presented a preoperative KPS of 70 or below (*p* < 0.0001) (Table 4). Although the conventional white-light group showed a 31.5% significant change in postoperative KPS over 70 from a preoperative KPS of 70 or below (*p*-value), the 5-ALA group resulted in a smaller amount in the same category of patients (12.5%, *p* = 0.083).

### 3.5. Cox Proportional Hazards Model—Survival Analysis

**Figure 3:**
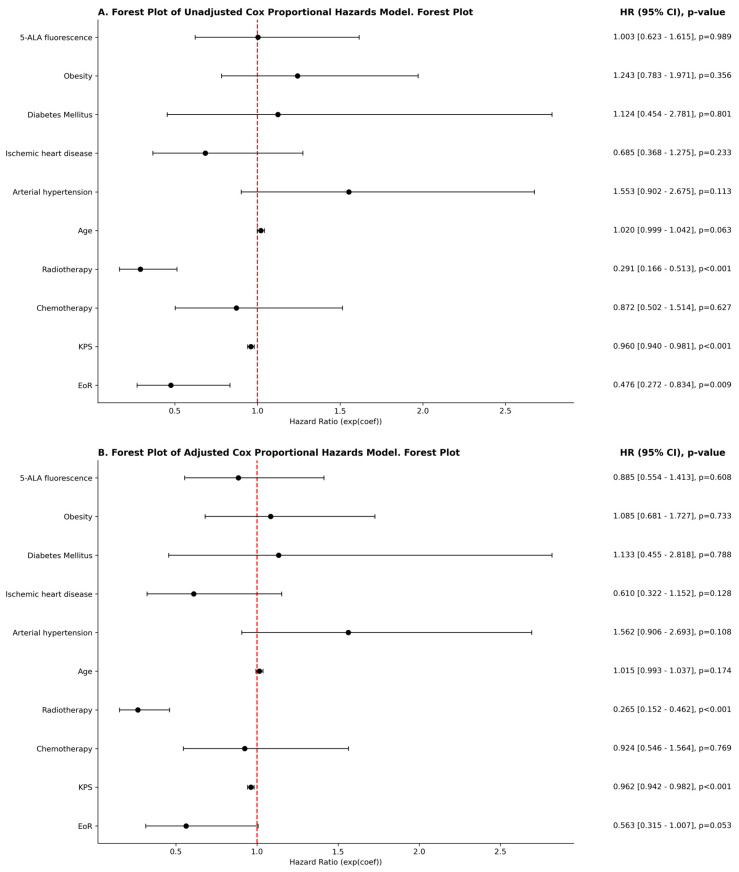
Proportional hazards model. Unadjasted (**A**) and Adjusted (B).

The Cox proportional hazards analysis revealed key predictors of overall survival. In the unadjusted model (Figure 3A), radiotherapy (HR = 0.291, 95% CI: 0.166–0.513, *p* < 0.001), higher Karnofsky Performance Status (KPS) (HR = 0.960, 95% CI: 0.940–0.981, *p* < 0.001), and gross total resection (EoR) (HR = 0.476, 95% CI: 0.272–0.834, *p* = 0.009) were significantly associated with improved survival. 5-ALA treatment, however, had no significant impact on survival (HR = 1.003, 95% CI: 0.623–1.615, *p* = 0.989).

In the adjusted model (Figure 3B), radiotherapy remained a strong protective factor (HR = 0.265, 95% CI: 0.152–0.462, *p* < 0.001), along with KPS (HR = 0.962, 95% CI: 0.942–0.982, *p* < 0.001). Gross total resection showed a trend toward improved survival (HR = 0.563, 95% CI: 0.315–1.007, *p* = 0.053), though it did not reach statistical significance. 5-ALA remained non-significant in the multivariable model (HR = 0.885, 95% CI: 0.554–1.413, *p* = 0.608), suggesting that while it improves resection extent, it does not independently affect survival when accounting for other clinical variables.

### 3.6. Case Examples

**Figure 4:**
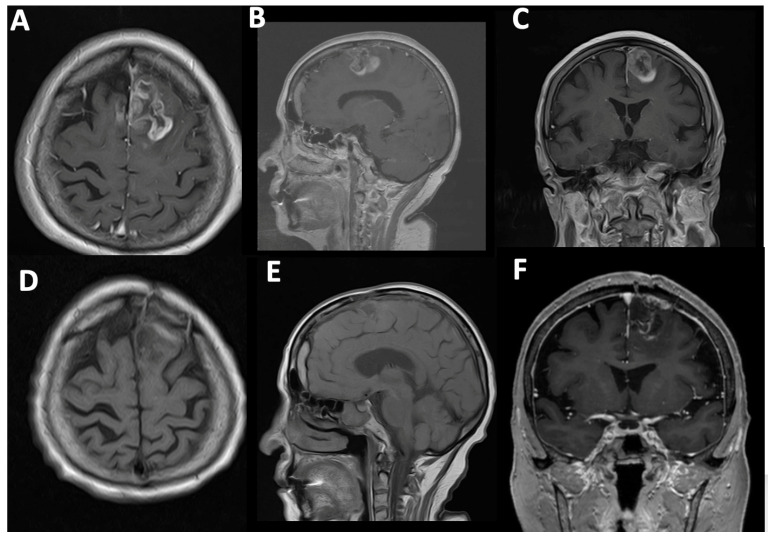
Fluorescent guided surgery. (**A**) A 54-year-old female first presented with a stroke suspicion, but after a detailed investigation, a left frontal HGG with relevant mass effect was revealed. Before the operation, the relatives also noticed signs of memory loss. Neurological examination revealed right-sided hemiparesis. (**A**–**C**) Preoperative T1-weighted contrast-enhancing images showing left frontal HGG. (**D**–**F**) Postoperative T1-weighted contrast-enhancing images showing no contrast enhancement. (**B**,**E**) Note the chronic subdural hematoma.

Case#1. A 73-year-old female with a medical history of arterial hypertension and diabetes mellitus presented with progressive headaches, dizziness, and cognitive decline. Family members also reported noticeable memory impairment. Neurological examination revealed motor aphasia and right-sided hemiparesis with a muscle strength of 4/5. The patient was alert with a Glasgow Coma Scale (GCS) score of 15 and had a Karnofsky Performance Status (KPS) of 70%. Preoperative contrast-enhanced MRI demonstrated a contrast-enhancing mass in the left frontal lobe with midline shift and mass effect, consistent with a high-grade glioma (Figure 4A–C). A chronic subdural hematoma was also noted in the ipsilateral hemisphere. The patient underwent successful resection via 5-ALA fluorescence-guided surgery. Intraoperative visualization under blue 400 nm light enabled maximal tumor delineation. Postoperative MRI confirmed complete removal of the contrast-enhancing component, with no residual visible tumor (Figure 4D–F).

**Figure 5:**
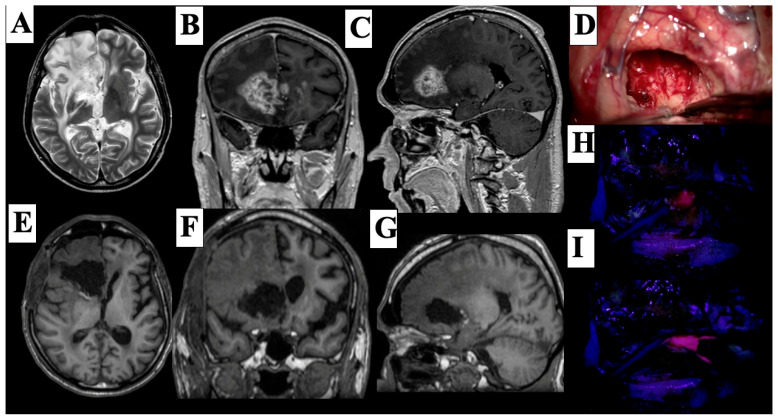
(**A**–**C**) Preoperative contrast-enhanced MRI showing a right frontal high-grade glioma with corpus callosum infiltration. (**D**) Intraoperative image after right frontotemporal craniotomy. (**E**–**G**) Postoperative MRI confirming gross total resection. (**H**,**I**) Intraoperative fluorescence images demonstrating a bright pink tumor under blue 400 light during 5-ALA-guided resection.

Case#2. A 57-year-old male with a known diagnosis of high-grade glioma presented with worsening neurological symptoms, including persistent headaches, dizziness, progressive cognitive impairment, and left-sided hemiparesis. He had previously undergone radiotherapy in 2022 with a total dose of 50 Gy targeting the primary lesion. Follow-up MRI in 2024 demonstrated radiological evidence of disease progression in the right frontal lobe extending into the corpus callosum (Figure 5A–C). The patient subsequently underwent a right frontotemporal craniotomy (Figure 5D). Intraoperative use of 5-aminolevulinic acid (5-ALA) allowed for fluorescence-guided resection of the lesion. Upon reaching the subcortical region, a vividly fluorescent pink tumor nodule was visualized under blue 400 nm filter light (Figure 5H,I), aiding in achieving gross total resection. Postoperative MRI confirmed the absence of contrast-enhancing residual tumor (Figure 5E–G).

## 4. Discussion

Our findings suggest that although 5-ALA use significantly increases the extent of resection, it does not independently improve overall survival. Kaplan–Meier analysis showed no significant survival difference between the 5-ALA and conventional white-light groups both before and after propensity score matching (log-rank *p* = 0.4993 and *p* = 0.7770, respectively). These results imply that differences in the tumor location, underlying biology, and access to adjuvant treatments may confound the survival benefits attributed to 5-ALA. High-grade gliomas (HGGs) remain a major therapeutic challenge due to their infiltrative behavior and dismal prognosis [1,2,3,4,5]. This study provides real-world data from a tertiary neurosurgical center in Kazakhstan, comparing fluorescence-guided surgery using 5-ALA with conventional white-light resection. The findings contribute to the ongoing discourse on the critical role of the extent of resection (EoR) in determining survival and functional outcomes in patients with HGGs [8,10,11,12].

In our study, Kaplan–Meier survival analysis revealed no significant difference in overall survival between the 5-ALA and conventional white-light groups following propensity score matching (log-rank *p* = 0.7770). However, patients who achieved gross total resection (GTR) demonstrated significantly improved survival compared to those with subtotal resection (log-rank *p* = 0.0423), reinforcing the prognostic relevance of maximal tumor removal. These findings align with the growing body of literature emphasizing the importance of the extent of resection in glioma outcomes. De Simone et al. [13] presented survival data on a molecularly defined cohort of IDH-mutant astrocytomas of the posterior cranial fossa, reporting favorable long-term outcomes with multimodal treatment. In their series, the majority of patients were WHO grade 2 or 3, with a small fraction being grade 4, and the mean follow-up exceeded 45 months. Although our cohort focused on IDH-wildtype high-grade gliomas (predominantly glioblastoma), the principle that extensive resection correlates with improved survival remains consistent across classifications.

Fluorescence-guided surgery using 5-aminolevulinic acid (5-ALA) has become an integral tool in the surgical management of high-grade gliomas (HGGs), with multiple studies confirming its utility in increasing the extent of resection (EOR) and, in select cohorts, prolonging survival. Our study reinforces these findings in part: the 5-ALA group demonstrated significantly higher gross total resection (GTR) rates (71.25% vs. 28.75%, *p* = 0.0002) that are consistent with previous reports such as those by Lavrador et al. (2024) [14], Lombardi et al. [15], and Eatz et al. [16], all of which associate 5-ALA with improved tumor visualization and greater surgical efficacy.

Our analysis confirms that 5-ALA fluorescence-guided surgery significantly increases gross total resection (GTR) rates; however, it does not independently improve overall survival when adjusting for confounders using propensity score matching. Notably, GTR was significantly associated with better survival (*p* = 0.0423), and our Cox regression analysis identified radiotherapy and Karnofsky Performance Status (KPS) as strong independent predictors of outcome. These results are consistent with those of Lombardi et al. [15] and Eatz et al. [16], who emphasize that 5-ALA enhances resection quality but does not replace the need for comprehensive oncological management. In our resource-limited setting, the survival benefit of 5-ALA may be masked by several factors, including limited access to awake craniotomy, intraoperative mapping, molecular profiling (MGMT, IDH), and standardized follow-up. Thus, while 5-ALA supports maximal safe resection, its full survival benefit likely depends on integration within a well-resourced, multidisciplinary treatment framework.

Despite ongoing advances, glial tumors remain enigmatic in their behavior, infiltration patterns, and therapeutic responsiveness. While gross total resection (GTR) is repeatedly associated with improved outcomes, achieving this goal—particularly in eloquent or deep-seated regions—requires more than surgical technique alone. As De Simone et al. stress, maximal resection must always be balanced with preservation of neurological function, especially in anatomically sensitive zones such as the posterior cranial fossa. This concept of onco-functional balance becomes even more critical in environments where intraoperative mapping or awake monitoring are not widely accessible [13].

Improved outcomes increasingly depend on the synergistic use of adjunct technologies. Tools like 5-ALA fluorescence enable more aggressive resections while helping preserve function, but their full benefit emerges only when integrated with complementary strategies. For example, combining 5-ALA with intraoperative mapping led to GTR in 96% of cases and significantly prolonged survival [16]. Molecular profiling, including identifying IDH mutations, MGMT methylation, EGFR amplification, further refines surgical decision making, enhancing patient selection for these tools and guiding postoperative therapy [4,13].

Still, the picture remains incomplete. Even with MRI-confirmed GTR, residual intraoperative fluorescence has been shown to predict poorer outcomes, suggesting that 5-ALA may highlight infiltrative tumor cells beyond what imaging can detect [17,18]. This underlines the importance of multimodal integration—not just surgical but molecular, functional, and imaging-guided techniques—to address the inherently diffuse nature of high-grade gliomas. While some studies question whether intraoperative MRI meaningfully improves results when added to 5-ALA [19], other findings support the combined use of mapping and neuromonitoring to maximize both the extent of resection and postoperative performance status [20,21].

Thus, while no single modality is sufficient, collectively, these techniques can help unlock better outcomes in a disease still shrouded in uncertainty. 5-ALA should not be viewed as a standalone solution but as one component of a broader oncological strategy that embraces precision, personalization, and multidisciplinary synergy [2,22,23].

While our findings confirm previously established evidence that gross total resection (GTR) is a key prognostic factor in HGG management, the value of our study lies in real-world verification within a resource-limited healthcare system. Specifically, we demonstrate that even with limited access to intraoperative mapping, molecular profiling, and advanced adjuvant protocols, 5-ALA significantly facilitates GTR in daily practice. This reinforces its value not only in high-resource centers but also in settings with infrastructural constraints.

## 5. Limitations

This study has several limitations that must be acknowledged. First, its retrospective, single-center design inherently carries risks of selection and documentation bias. Treatment allocation—whether patients received 5-ALA-guided or conventional white-light resection—was not randomized but instead determined by surgeon preference and resource availability, introducing potential selection bias. Second, critical molecular prognostic markers such as MGMT promoter methylation and IDH mutation status were missing for a substantial proportion of patients, precluding molecular subgroup analysis and limiting genomic risk stratification. Third, the absence of intraoperative brain mapping and awake craniotomy constrained the ability to achieve maximal safe resection in eloquent regions, particularly in complex or high-risk anatomical areas.

Additionally, inconsistencies in adjuvant therapy access and compliance, variable follow-up durations, and incomplete recurrence documentation—common limitations in resource-constrained and emerging healthcare systems—may have influenced the observed survival outcomes. These limitations reflect the challenges of translating clinical trial protocols into real-world practice. Nevertheless, this study offers valuable insights into the practical utility of 5-ALA fluorescence-guided surgery in a non-trial setting, emphasizing the need for multidisciplinary infrastructure, molecular diagnostics, and standardized treatment pathways to fully realize its benefits.

## 6. Conclusions

This study confirms that 5-ALA fluorescence-guided surgery significantly increases the rate of gross total resection in patients with high-grade gliomas, particularly in tumors located in the temporal and parietal lobes. Although patients in the 5-ALA group demonstrated better survival trends within the first 50 months postoperatively, adjusted analyses—including propensity score matching and Cox regression—did not show a statistically significant survival advantage. Instead, survival was independently associated with the extent of resection, postoperative functional status, and receipt of radiotherapy. These findings reinforce the value of 5-ALA as a surgical adjunct that enhances tumor resection but suggest that its survival benefit is not standalone and depends on integration with adjuvant therapy and comprehensive oncologic care.

Although the association between GTR and improved survival is well-documented, our findings validate the applicability of 5-ALA-assisted surgery in a real-world, resource-limited neurosurgical setting. The study highlights the critical need for integrated oncological support to fully realize the potential benefits of fluorescence-guided resection.

Future prospective and multicenter studies incorporating molecular data and standardized treatment protocols are warranted to better define the long-term benefits of 5-ALA within modern neuro-oncological care pathways.

## Figures and Tables

**Table 2:** Pre- and postoperative KPS and tumor characteristics.

Preoperative KPS > 70	5-ALA	White-Light Group	Total	*p*-Value	Statistical Test
≤70	58 (82.86%)	59 (83.10%)	117	0.9696	Chi-square
>70	12 (17.14%)	12 (16.90%)	24		
**Postoperative KPS > 70**					
≤70	39 (55.71%)	49 (69.01%)	88	0.103	Chi-square
>70	31 (44.29%)	22 (30.99%)	53		
**Re-surgery**					
No	62 (88.57%)	59 (83.10%)	121	0.3517	Chi-square
Yes	8 (11.43%)	12 (16.90%)	20		
**Therapy after surgery**					
No	66 (94.29%)	67 (94.37%)	133	1	Fisher’s Exact
Yes	4 (5.71%)	4 (5.63%)	8		
**IDH1**					
No	37 (60.66%)	53 (80.30%)	90	0.0149	Chi-square
Yes	24 (39.34%)	13 (19.70%)	37		

**Table 4:** Preoperative vs. postoperative KPS.

	Postoperative KPS ≤ 70	Postoperative KPS > 70	Total	McNemar’s *p*-Value
	**Total Sample**			
Preoperative KPS ≤ 70	82 (58.16%)	35 (24.82%)	117	<.0001
Preoperative KPS > 70	6 (4.26%)	18 (12.77%)	24	
Total	88	53	141	
Conventional white-light group	**Conventional white-light group**			
Preoperative KPS ≤ 70	36 (51.43%)	22 (31.43%)	58	0.0001
Preoperative KPS > 70	3 (4.29%)	9 (12.86%)	12	
Total	39	31	70	
	**5-ALA group**			
Preoperative KPS ≤ 70	46 (64.79%)	13 (18.31%)	59	0.0124
Preoperative KPS > 70	3 (4.23%)	9 (12.68%)	12	
Total	49	22	71	

## Data Availability

All data is available upon request from the corresponding author.

## Supplementary Materials

The following supporting information can be downloaded at: https://www.mdpi.com/article/10.3390/cancers17121897/s1, File S1: 5-ALA and Control group dataset

## References

[1] Hadjipanayis C.G., Widhalm G., Stummer W. (2015). What is the Surgical Benefit of Utilizing 5-Aminolevulinic Acid for Fluorescence-Guided Surgery of Malignant Gliomas?. Neurosurgery.

[2] De Simone M., Iaconetta G., Palermo G., Fiorindi A., Schaller K., De Maria L. (2024). Clustering Functional Magnetic Resonance Imaging Time Series in Glioblastoma Characterization: A Review of the Evolution, Applications, and Potentials. Brain Sci..

[3] Palmieri G., Cofano F., Salvati L.F., Monticelli M., Zeppa P., Di Perna G., Melcarne A., Altieri R., La Rocca G., Sabatino G. (2021). Fluorescence-Guided Surgery for High-Grade Gliomas: State of the Art and New Perspectives. Technol. Cancer Res. Treat..

[4] De Simone M., Conti V., Palermo G., De Maria L., Iaconetta G. (2024). Advancements in Glioma Care: Focus on Emerging Neurosurgical Techniques. Biomedicines.

[5] Altieri R., Raimondo S., Tiddia C., Sammarco D., Cofano F., Zeppa P., Monticelli M., Melcarne A., Junemann C., Zenga F. (2019). Glioma surgery: From preservation of motor skills to conservation of cognitive functions. J. Clin. Neurosci..

[6] Altieri R., Hirono S., Duffau H., Ducati A., Fontanella M.M., La Rocca G., Melcarne A., Panciani P.P., Spena G., Garbossa D. (2020). Natural history of de novo high grade glioma: First description of growth parabola. J. Neurosurg. Sci..

[7] Stummer W. (2016). Factors confounding fluorescein-guided malignant glioma resections: Edema bulk flow, dose, timing, and now: Imaging hardware?. Acta Neurochir..

[8] Mirza A.B., Christodoulides I., Lavrador J.P., Giamouriadis A., Vastani A., Boardman T., Ahmed R., Norman I., Murphy C., Devi S. (2021). 5-Aminolevulinic acid-guided resection improves the overall survival of patients with glioblastoma-a comparative cohort study of 343 patients. Neurooncol. Adv..

[9] Xiao Y., Li M., Wang X., Tan J., Qin C., Liu Q. (2024). Fluorescein-guided surgery in high-grade gliomas: Focusing on the eloquent and deep-seated areas. J. Cancer Res. Clin. Oncol..

[10] Hegi M.E., Diserens A.-C., Gorlia T., Hamou M.-F., De Tribolet N., Weller M., Kros J.M., Hainfellner J.A., Mason W., Mariani L. (2005). MGMT gene silencing and benefit from temozolomide in glioblastoma. N. Engl. J. Med..

[11] Koekkoek J.A.F., van der Meer P.B., Pace A., Hertler C., Harrison R., E Leeper H., A Forst D., Jalali R., Oliver K., Philip J. (2023). Palliative care and end-of-life care in adults with malignant brain tumors. Neuro Oncol..

[12] Lacroix M., Abi-Said D., Fourney D.R., Gokaslan Z.L., Shi W., DeMonte F., Lang F.F., McCutcheon I.E., Hassenbusch S.J., Holland E. (2001). A multivariate analysis of 416 patients with glioblastoma multiforme: Prognosis, extent of resection, and survival. J. Neurosurg..

[13] De Simone M., Choucha A., Ranalli C., Pecoraro G., Appay R., Chinot O.L., Dufour H., Iaconetta G. (2025). Astrocytomas IDH-mutant of posterior cranial fossa, clinical presentation, imaging features and onco-functional balance in surgical management. Neurosurg. Rev..

[14] Lavrador J.P., Marchi F., Elhag A., Kalyal N., Mthunzi E., Awan M., Wroe-Wright O., Díaz-Baamonde A., Mirallave-Pescador A., Reisz Z. (2024). In Situ Light-Source Delivery During 5-Aminulevulinic Acid-Guided High-Grade Glioma Resection: Spatial, Functional and Oncological Informed Surgery. Biomedicines.

[15] Lombardi G., Barresi V., Castellano A., Tabouret E., Pasqualetti F., Salvalaggio A., Cerretti G., Caccese M., Padovan M., Zagonel V. (2020). Clinical Management of Diffuse Low-Grade Gliomas. Cancers.

[16] Eatz T.A., Eichberg D.G., Lu V.M., Di L., Komotar R.J., Ivan M.E. (2022). Intraoperative 5-ALA fluorescence-guided resection of high-grade glioma leads to greater extent of resection with better outcomes: A systematic review. J. Neurooncol..

[17] Coburger J., Wirtz C.R. (2019). Fluorescence guided surgery by 5-ALA and intraoperative MRI in high grade glioma: A systematic review. J. Neurooncol..

[18] Mirza A.B., Vastani A., Suvarna R., Rashed S., Al-Omari A., Mthunzi E., Fayez F., Rampersad N., Jung J., Baamonde A.D. (2025). Preoperative and intraoperative neuromonitoring and mapping techniques impact oncological and functional outcomes in supratentorial function-eloquent brain tumours: A systematic review and meta-analysis. EClinicalMedicine.

[19] Gandhi S., Tayebi Meybodi A., Belykh E., Cavallo C., Zhao X., Syed M.P., Borba Moreira L., Lawton M.T., Nakaji P., Preul M.C. (2019). Survival Outcomes Among Patients With High-Grade Glioma Treated With 5-Aminolevulinic Acid-Guided Surgery: A Systematic Review and Meta-Analysis. Front Oncol..

[20] Acerbi F., Broggi M., Schebesch K.-M., Höhne J., Cavallo C., De Laurentis C., Eoli M., Anghileri E., Servida M., Boffano C. (2018). Fluorescein-guided surgery for resection of high-grade gliomas: A multicentric prospective phase II study (FLUOGLIO). Clin. Cancer Res..

[21] Schucht P., Beck J., Abu-Isa J., Andereggen L., Murek M., Seidel K., Stieglitz L., Raabe A. (2012). Gross total resection rates in contemporary glioblastoma surgery: Results of an institutional protocol combining 5-aminolevulinic acid intraoperative fluorescence imaging and brain mapping. Neurosurgery.

[22] Coburger J., Hagel V., Wirtz C.R., König R. (2015). Surgery for Glioblastoma: Impact of the Combined Use of 5-Aminolevulinic Acid and Intraoperative MRI on Extent of Resection and Survival. PLoS ONE.

[23] Aldave G., Tejada S., Pay E., Marigil M., Bejarano B., Idoate M.A., Díez-Valle R. (2013). Prognostic value of residual fluorescent tissue in glioblastoma patients after gross total resection in 5-aminolevulinic Acid-guided surgery. Neurosurgery.

